# The efficacy of soap against schistosome cercariae: A systematic review

**DOI:** 10.1371/journal.pntd.0010820

**Published:** 2022-10-03

**Authors:** Jiaodi Zhang, Ana K. Pitol, Laura Braun, Lucinda Hazell, Michael R. Templeton

**Affiliations:** 1 Department of Civil and Environmental Engineering, South Kensington Campus, Imperial College London, London, United Kingdom; 2 Department of Vector Biology, Liverpool School of Tropical Medicine, Liverpool, United Kingdom; 3 Department of Disease Control, London School of Hygiene Tropical Medicine, London, United Kingdom; Federal University of Agriculture Abeokuta, NIGERIA

## Abstract

**Background:**

Schistosomiasis is a parasitic disease that is endemic in 78 countries and affects almost 240 million people worldwide. It has been acknowledged that an integrated approach that goes beyond drug treatment is needed to achieve control and eventual elimination of the disease. Improving hygiene has been encouraged by World Health Organisation, and one aspect of good hygiene is using soap during water-contact activities, such as bathing and doing laundry. This hygiene practice might directly reduce the skin exposure to cercariae at transmission sites. A systematic review was carried out to investigate the efficacy of soap against schistosome cercariae and to identify the knowledge gaps surrounding this topic.

**Methodology:**

Six online databases were searched between 5^th^ and 8^th^ July of 2021. Records returned from these databases were screened to remove duplicates, and the remaining records were classified by reading titles, abstracts, and full texts to identify the included studies. The results were categorised into two groups based on two different protective mechanisms of soap (namely, damage to cercariae and protection of skin).

**Conclusions:**

Limited research has been conducted on the efficacy of soap against schistosome cercariae and only 11 studies met the criteria to be included in this review. The review demonstrates that soap has the potential of protecting people against schistosome cercariae and there are two protective aspects: (1) soap affects cercariae adversely; (2) soap on the skin prevents cercariae from penetrating the skin, developing into adult worms and producing eggs. Both aspects of protection were influenced by many factors, but the differences in the reported experimental conditions, such as the cercarial endpoint measurement used and the cercaria numbers used per water sample, lead to low comparability between the previous studies. This review indicates that more evidence is needed to inform hygiene advice for people living in schistosomiasis endemic areas.

## Author summary

Schistosomiasis affects millions of people living in low- and middle-income countries lacking safe access to water, sanitation and hygiene (WASH). Infection occurs through skin-contact with water containing schistosome cercariae which are released from the snail intermediate hosts. The disease is mainly controlled through drug treatment with praziquantel in humans, but reinfection is possible if contact with cercaria-contaminated water continues. Despite all the efforts that have been made, this disease remains a major public health problem in disease-endemic regions. The use of soap during water contact, as part of good hygiene practice, may be an effective complementary control method as it might reduce the penetration of schistosome cercariae into the skin, thereby reducing the likelihood of reinfection. We conducted a systematic review to summarise previous research into the efficacy of soap against schistosome cercariae, and to identify current knowledge gaps to inform future studies. The information gathered from this review can provide policymakers with preliminary scientific evidence (e.g. the soap types, soap amounts, exposure times that are needed) to further emphasise the importance of effective soap usage in reducing the spread of schistosomiasis.

## Introduction

Schistosomiasis is a parasitic disease caused by trematode worms of the genus *Schistosoma* that affects approximately 240 million people worldwide [1]. It is estimated that the global burden of schistosomiasis is 1.6 million disability-adjusted life years (DALYs) according to the Global Burden of Disease Study 2019 [2]. This disease occurs because of skin-contact with water contaminated by cercariae, which are a larval stage of the schistosome parasite. The life cycle of the parasite involves the excretion of *Schistosoma* eggs into freshwater via the faeces or urine of infected individuals depending on the *Schistosoma* species. The eggs then hatch into miracidia which infect the intermediate hosts, specific freshwater snails. The compatible infected snails passing the prepatent period release cercariae, which can penetrate human skin, develop into adult worms and produce eggs inside humans.

Several interventions can be adopted to control schistosomiasis transmission, such as preventive chemotherapy and snail control. Preventive chemotherapy, namely mass drug administration with praziquantel (Merck KGaA), has been the primary control measure in the last few decades since it is cost-effective and safe [1,3,4]. However, this intervention cannot prevent reinfection which is likely in communities relying on contaminated freshwater for daily water activities. The mass drug administration also relies heavily on the coverage level of drug distribution and ongoing financial support [5,6]. Snail control using molluscicides has also proven to be an effective way to interrupt disease transmission and reduce disease prevalence [7–9]. Nevertheless, there are concerns that molluscicidal chemicals might adversely affect aquatic lifeforms, and some studies have shown that mollusciciding is labour-intensive and difficult to apply effectively in some settings [10].

Despite the efforts and progress made, schistosomiasis remains a major public health problem in disease-endemic regions. In the new road map for neglected tropical diseases, the World Health Organisation (WHO) has proposed a target of eliminating schistosomiasis as a public health problem by 2030, aiming to reduce the proportion of heavy intensity infections to less than 1% [11]. In the World Health Assembly (WHA) resolution 65.21, water, sanitation and hygiene (WASH) interventions are encouraged as components of schistosomiasis control and elimination in disease-endemic areas [12], emphasising the importance of WASH as part of a multi-faceted and sustainable disease control strategy [13,14].

Hygiene can be defined as personal and household-level practices (e.g. handwashing, bathing, laundry) which are aimed at achieving cleanliness, maintaining health and preventing disease [6,11]. One aspect of good hygiene is the use of soap when washing, and this hygienic practice is important to prevent many infectious diseases [15,16], such as diarrhea caused by enteric pathogens [17], trachoma [18] and acute respiratory diseases [19]. Apart from soap, alternative handwashing materials such as ash can be used. However, it remains unclear whether the use of ash during handwashing is effective at protecting people from viral and bacterial infections [20].

The use of soap might play a vital role in controlling schistosomiasis and Grimes *et al*. have suggested that this role might have two aspects [21]. The first aspect is that people who are using soap during water contact may be protected from infection. The second aspect is that soap may kill other schistosome life stages in water, including miracidia, cercariae and snails, and thereby help prevent other people from becoming infected. Since infection only occurs when cercariae penetrate human skin during water-contact activities and several of these common water-contact activities such as laundry, bathing and handwashing involve the use of soap, it is crucial to understand the interaction between soap use and cercaria infectivity.

Some field studies have been carried out in disease-endemic regions to investigate the effectiveness of soap use in preventing schistosomiasis, but the results remain inconclusive, since some observed a positive effect and some did not. Endod (*Phytolacca dodecandra*) berries are a typical local soap in Ethiopia, and these small berries can be used to wash clothes after being grounded either in dried or fresh condition and placed in water [22]. One field study in Ethiopia reported a significant reduction in the prevalence of *S*. *mansoni* infection in females after soap containing ground Endod berries was distributed to people washing clothes, though the prevalence in males increased insignificantly [23]. This might be because the majority of washing activities are likely to be performed by women and girls [21]. The result of another study suggested the possible positive influence of soap, as this study found no significant association between clothes-washing and *S*. *haematobium* infection, with a high reported soap-use rate of 98.4% during laundry in the rural communities of Zanzibar [24]. However, there are other studies which did not show that soap has any positive protective effect against schistosomiasis. A field study in a mixed *S*. *haematobium—S*. *mansoni* focus in Niger found that the use of soap did not prevent children from being infected, though almost all women claimed to use soap when washing their children at the transmission sites [25]. Also, no significant relationship was found between both *S*. *mansoni* and *S*. *haematobium* infection and the use of handwashing facilities equipped with available soap and water [26,27]. These conflicting results suggest that it is not clear whether the use of soap can be an effective intervention, or that the details of the mode of soap use (e.g. soap type, soap concentration, exposure time) are responsible for the varying reported results. The effectiveness of using soap probably varies significantly since the protective effect of soap is highly human behaviour-specific [21], in terms of the different practices of using soap, and the disease prevalence can also be influenced by many non-hygiene related factors. Therefore, results obtained from controlled experiments are likely to be more informative about the efficacy of soap against schistosome cercariae than observational field studies since these controlled experiments remove the potential influences of human behaviour and non-hygiene related factors.

The increased use of soap has the potential to be adopted as an effective intervention to prevent schistosomiasis. This research aims to summarise the knowledge obtained from previous experiments in terms of soap use against schistosome cercariae and identify knowledge gaps. The information gathered from this research will help to optimise soap use from a disease prevention viewpoint and form the basis of hygiene practice guidelines (e.g. the soap types, soap amounts, exposure times) to control the spread of schistosomiasis.

## Methods

Our systematic review followed the guidelines of the Preferred Reporting Items for Systematic reviews and Meta-Analyses (PRISMA). This review was not registered, and the protocol was not prepared. Papers were searched between 5^th^ and 8^th^ July of 2021 in six databases: Web of Science, Scopus, PubMed, The British Library, Google Scholar, and China National Knowledge Infrastructure (CNKI). All languages and document types were included, and databases were searched from inception to the present day. All databases were searched for any combination of common schistosomiasis-related terms (Schistosom*, Cercari*, *mansoni*, *haematobium*, *japonicum*, Bilharz*, Snail fever) and hygiene-related terms (Soap*, Detergent*, Endod, *Phytolacca dodecandra*, Ash, Laund*, Bath*, Shower*, Handwash*, Clean*, Wash*, Hygien*) in the title. A full list of search terms and search strategies for each database can be found in S1 Methods. Previous studies have investigated the efficacy of soap as well as plant extracts against cercariae. This systematic review does not include individual plant extracts for the following reasons: (1) the scope of this review is to understand the efficacy of soap that people currently use for hygiene purposes; (2) it is uncertain whether those plant extracts can be added into soap products; (3) the toxicity of the plant extracts against cercariae is likely different when they are mixed with different components. For example, one study found that the protection of soaps containing essential oils on mouse tails varied from 0% to 100% when different concentrations of the same essential oils were mixed with different proportions of other materials [28]. Therefore, we only included studies of products that people have used for hygiene purposes (i.e. soaps). However, we did include endod because endod berries have traditionally been used in Ethiopia as soap and are known to provide protection against schistosome cercariae [22].

### Classification criteria

**Fig 1:**
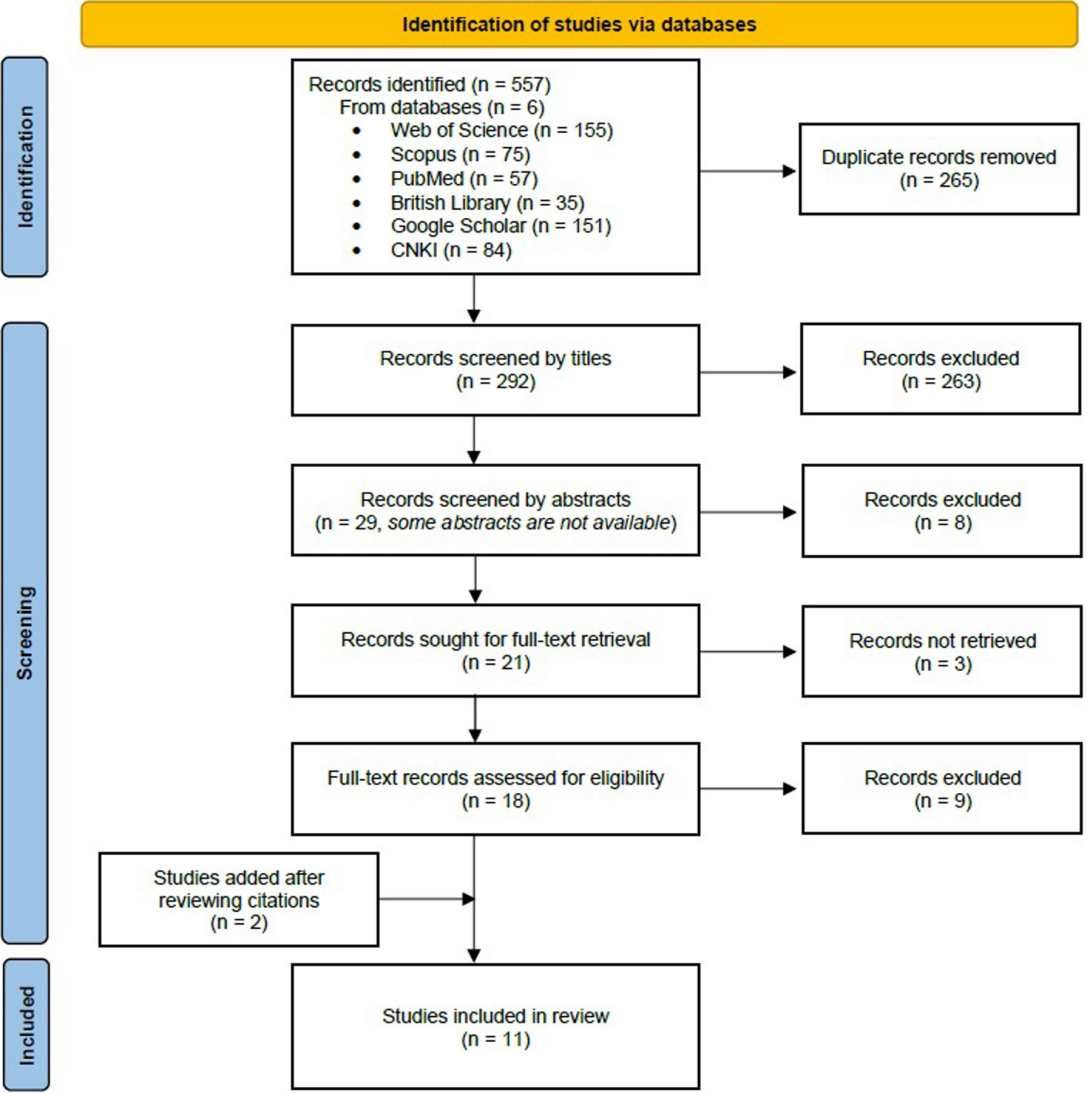
PRISMA flow diagram.

A classification system was developed to screen and classify the records. First, duplicate records were removed and assigned the code 1. Remaining records were classified by screening titles, and when the titles were not relevant to the scope of this review, the records were removed and assigned the code 2. Afterwards, the records were classified by reading their abstracts. Records were excluded and assigned the codes 3–5 when their abstracts showed the studies were (1) not experiment-based studies (2) not about the use of soap (3) not about the effect of soap against cercariae. The full texts of the remaining records were read. When the papers met the same criteria for screening abstracts, those papers were assigned the codes 6–8. Chapter titles of books were screened; however no relevant studies were found. The remaining papers were included in this review and assigned the codes 9–10 based on the two protective aspects of soap. Additional studies which were referenced in the already included papers in a way indicating that these studies met the objective of this review were also retrieved for the full texts to be read, included and assigned the corresponding codes. A flow diagram (Fig 1) were adapted from the PRISMA 2020 flow diagram with minor changes to summarise the classification process. The classification results including the classification process, codes and a list of the excluded and included records are summarised in the S1 Table.

Full texts were acquired from Imperial College London Library and the British Library. Non-English papers were translated using Google Translate first to decide whether the study should be included. Then, translations of those included papers were checked by native speakers.

### Data extraction

Two tables (S1 Table) were developed based on two aspects of potential protection from soap: an adverse effect of soap on cercariae, and the protection on skin against cercariae. The first two authors (JZ and AKP) independently extracted data on potential protection from soap (S1 Table). Any discrepancies in the extracted data between the two authors were discussed and resolved.

Some washing powders and detergents were tested in the included papers. These are described as powder soap in the sections below.

### Synthesis of results and data analysis

**Fig 2:**
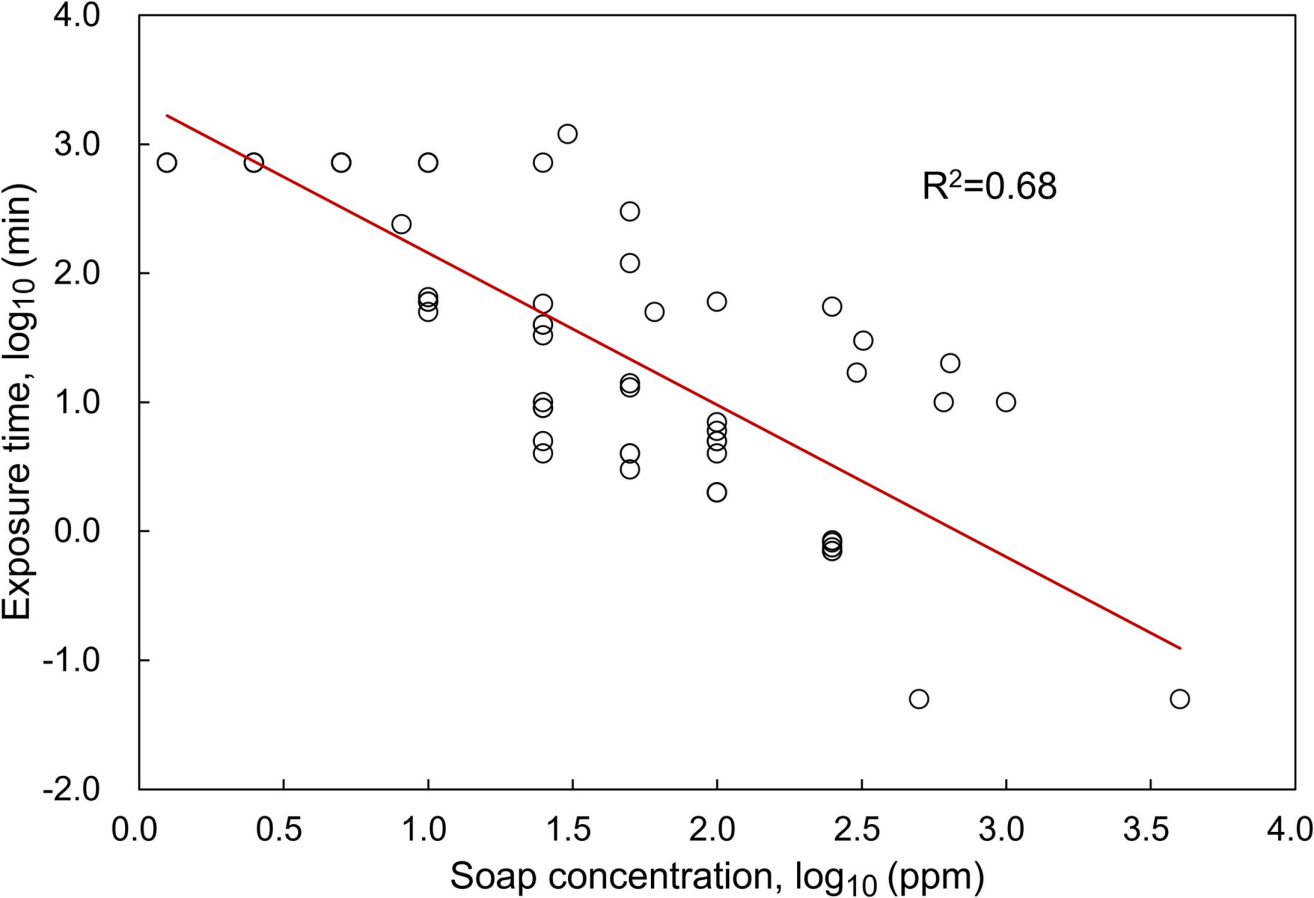
Soap concentrations and exposure times required to achieve 100% cercaria mortality [34,37,38,42,44]. Data are generated from soaps which were tested in five studies. *S*. *haematobium* cercariae were used in the study by Lemma [42], while *S*. *mansoni* were used in the other four studies. Several assumptions were made as follows: (1) the study by Mimpfoundi & Dupouny stated that the cercariae survived more than 12 hours in some experimental conditions, and therefore it was assumed that cercariae only survived 12 hours in order to include this research [38]; (2) LC_90_ was used as the dose for 100% mortality in the study by Monkiedje, Anderson & Englande [34]; (3) only one individual compound existed in both soaps tested in the study by Pacheco & Jansen [44]; (4) in the studies by Mimpfoundi & Dupouny and Okwuosa & Osuala, the exposure time of “immediate cercaricidal effect/death” was assumed to be three seconds based on our estimation that at least three seconds are required to determine cercaria death [37,38]; (5) The concentrations in mg/L and mol/L were converted to ppm, assuming that the liquid density is 1 g/mL.

Parts per million (ppm) is used to compare the soap concentrations in the sections below, since the majority of soap concentrations in included papers were presented in ppm. The major assumption made in our data analysis is that the liquid density is 1 g/mL when converting concentrations in mg/L or mol/L to ppm. There are several other assumptions made to include the data about the effect of soap on cercaria mortality as follows (can be also found in the caption of Fig 2): (1) cercaria survival was restricted to only 12 hours in one study; (2) LC_90_ was used as the dose for 100% mortality in one study; (3) only one individual compound existed in both soaps tested in one study; (4) in two studies stating “immediate cercaricidal effect/death”, the exposure time was assumed to be three seconds based on our estimation that at least three seconds are required to determine cercarial death. Regarding the soap concentration in ppm during handwashing, a rough estimate was performed as below. One study has found that it takes 0.4–9 mL of liquid soap for each handwash [29]. According to the United Nations Children’s Fund, the volume of water consumed can be up to 2 L and down to 0.3–0.6 L per handwash, depending on whether or not the water is running when scrubbing the hands with soap [30]. Given the data above, it is estimated that the average soap concentration during handwashing can range from 200 ppm (0.4 mL / 2 L) to 30000 ppm (9 mL / 0.3 L).

The results of the two soap protective aspects were presented in different ways. For the effect of soap on cercariae, different measures (e.g. time taken to kill all cercariae at a concentration, worm burden, egg load) were used depending on the cercaria endpoint assessed and one model simulation for the effect on cercaria mortality was performed using SPSS software (Version 23, IBM). For the soap protection on skin against cercaria penetration, the protection which is the reduction in worms recovered from soap-treated mice/rats compared with the control was calculated to show the results. All the figures except Fig 1 were drawn using the Microsoft Excel (Version 2019).

## Results and discussion

The result of our classification process is shown in the flow diagram (Fig 1). A total of 557 records were returned and 292 records remained after duplicates were removed. After screening titles and abstracts, 21 records were sought for full-text retrieval including the records whose abstracts were not available and two books, while three full texts were not available. All the (sub)chapter titles of those two books were read and the full texts of the (sub)chapters were read when the titles showed they were relevant to the review, though no relevant studies in those books were found. Two additional studies which were referenced in already-included studies were also added to the review. In the end, 11 studies were included in the analysis of this systematic review.

### Soap type

Three major types of soap were investigated in the included studies: locally produced soap, commercial soap, and experimental soap (Table 1). Endod, a locally produced soap, was not only toxic to cercariae but also to miracidia, snails and fish [31–34]. Its molluscicidal activity has received extensive attention since this plant-derived molluscicide is argued to be more environmentally friendly and economical than synthetic molluscicides [23,33,35]. The protective effect of some commercial soaps (e.g. Dettol, Lux, Omo) was also explored in other studies [28,36–38]. In addition, two kinds of experimental soap formulations, Schistopel and soaps containing essential oils, were tested. Schistopel is an experimental soap formulation with hexachlorophene as a bacteriostatic agent [39], while other experimental soaps incorporated essential oils extracted from local plants into soap formulations [28,40]. Similar to endod, some essential oils showed promising toxicity against the intermediate hosts of *Schistosoma* spp. and could be potentially used as a molluscicide [41].

**Table 1:** Summary of the soaps considered in the included papers.

Soap type	Soap name	# of studies	Protective effect	Cercarial endpoint	Reference
Local soap	Endod	3	Effect on cercariae	Mortality^a^	[32,34,42]
				Motility^b^	[32]
				Viability^c^	[32]
	Local soap in Nigeria	1	Effect on cercariae	Mortality	[37]
Commercial soap	Omo	2	Effect on cercariae	Mortality	[37,38]
				Viability	[37]
	Elephant	1	Effect on cercariae	Mortality	[37]
	Surf	1			
	Action	1			
	Flash	1			
	Pax	1			[38]
	Genie	1			
	Sol	1			
	Eucalipto	1	Protection on skin	Infectivity^d^	[28]
	Lux	1		Viability	
	Dettol	1	Effect on cercariae	Mortality	[36]
				Motility	
				Morphology^e^	
		1	Protection on skin	Viability	
Experimental soap	Schistopel	2	Protection on skin	Infectivity	[43]
				Viability	[39,43]
	Soaps containing essential oils	2	Protection on skin	Infectivity	[28,40]
				Viability	
Others	Sodium oleate	1	Effect on cercariae	Mortality	[44]
	Sodium ricinoleate	1		Motility	

^a^Mortality = Cercaria’s survival percentage

^b^Motility = Cercaria’s ability to move

^c^Viability = Cercaria’s ability to penetrate the skin and develop into schistosomes [45]

^d^Infectivity = Cercaria’s ability to attach to or penetrate skin [45]

^e^Morphology = Cercaria’s shape

### Protective roles against cercariae

Based on the findings from the included studies in this review, the role of soap against schistosome cercariae has two aspects: (1) soap has adverse effects on cercariae; (2) soap gives protection against cercaria penetration into the skin, developing into adult worms and producing eggs. Different adverse endpoints of cercariae were assessed including mortality, motility, morphological change, infectivity and viability. For the first three endpoints, microscopes were the tool for observing cercariae. For infectivity and viability, mice/rats were exposed to cercariae by immersing their tails in water, and the percentage of cercaria penetration and the worm burden or other indices were determined after exposure.

### Protective role 1: The adverse effect of soap on cercariae

#### The adverse effect on cercaria mortality

The results suggest that soap is less toxic to schistosome cercariae than other chemicals, such as niclosamide and chlorine [34,46]. For example, the 50% lethal dose of niclosamide when treating cercariae for 4 hours was 3650 times lower than that associated with endod [34]. Commercial soap tended to be more toxic than local soap since the commercial ones needed less time to kill cercariae than the local Nigerian soap at the same concentration [37]. Endod also showed a less lethal effect than commercial soaps in two other studies [37,38,42].

As shown in Fig 2, the lethal effect of soap was associated with soap concentration and exposure time, and the log-transformed data can fit well to a linear regression model with a reasonable R^2^ of 0.68. The exposure time required to achieve 100% cercarial mortality is reduced with increasing soap concentration [37,38,42,44]. The soap’s toxicity might be also affected by other factors, such as water hardness; one study found that an increase in water hardness increased the cercaricidal effect of powder soaps [38].

#### The adverse effect of soap on cercaria motility and morphological change

The motility and morphology of cercariae can also be affected by exposure to soap. Pacheco and Jansen presented cercaria motility in three different phases including sinking to the bottom, slower movement and immobilization which was also used to determine death [44]. Even though some cercariae were not killed by soap, their swimming behaviours became less active [32,36] and this behavioural change was also associated with the concentration and exposure time [32]. Regarding cercaria morphology, only Edungbola reported significant morphological damage, including evaginated ventral suckers and irreversible severed tails from the body at the 10^−2^ dilution of Dettol which should mean that adding 1 part of Dettol to 99 parts of water [36].

#### The adverse effect of soap on cercaria viability

In studies examining the effect of soap on cercarial viability, cercariae exposed to different concentrations of soap diluted in water were used to infect mice via tail immersion, and worm burden and egg load were determined. Worm burden (load) and egg load refer to the worms and eggs recovered from hosts after exposure to cercariae [32,37]. Other indices were also measured in one study, including anaemia given by haemoglobin concentration (g/100 ml) and packed cell volume (% age), and hepatosplenomegaly [37].

**Fig 3:**
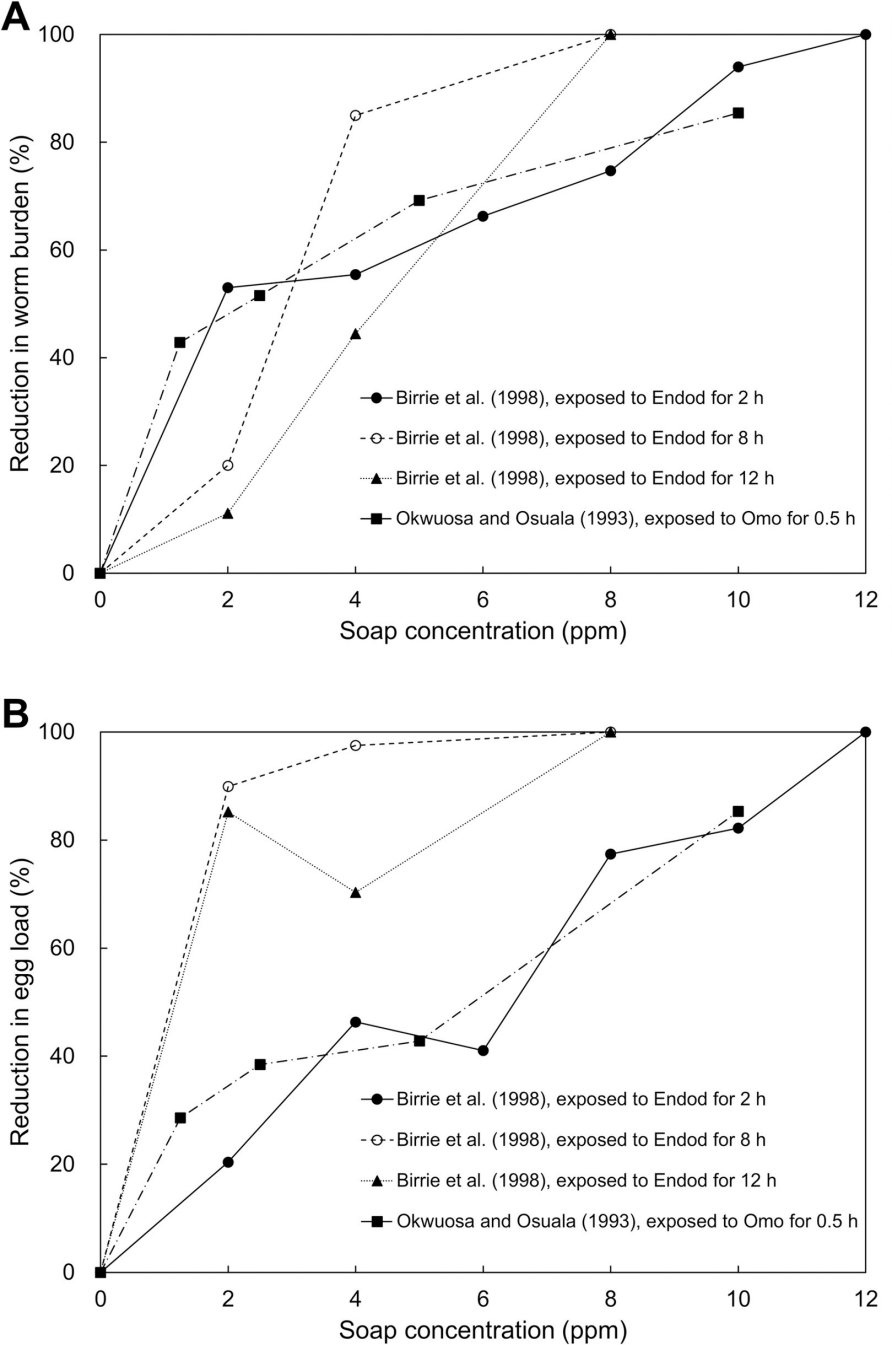
The reduction in worm burden (A) and egg load (B) under different soap treatment conditions [32,37]. The reduction in worm burden was calculated by comparing the average of worms recovered in the soap-treated group with that in the untreated group. The species in these two studies was *S*. *mansoni*.

Similar to was found in studies using cercariae mortality, where the impact of soap increased with soap concentration, both studies have demonstrated that the worm burden reduced with an increase in soap concentration (Fig 3A). Okwuosa and Osuala found Omo, a commercial powder soap, reduced about 85% of the worm burden and egg load at 10 ppm after treating for 30 minutes, compared to the untreated group [37]. Birrie *et al*. tested endod and this soap totally suppressed the cercarial viability at 12 ppm after treating for 2 hours, and the worm burden and egg load were also significantly reduced at lower concentrations (Fig 3) [32]. The result of this study also demonstrated that a lower soap concentration was needed to render all cercariae incapable of developing into adult worms at longer soap exposure times, indicating that soap use duration might also play an important role in the ability of soap to affect the viability of cercariae.

### Protective role 2: The protective effect of soap on rodent tails against schistosome cercariae

Another protective aspect of soap against schistosome cercariae is that soap could provide human skin with protection, preventing cercariae from penetrating into the skin, developing into adult worms and producing eggs. In previous studies, living mice or rats were used as the host and soap was applied on their tails before being exposed to cercariae. Cercariae infectivity and viability were the adverse endpoints assessed, as the percentage of cercariae that failed to penetrate was determined in some studies [28,40,43], and worms were recovered after exposure in all studies [28,36,39,40,43] and the eggs were collected in some studies [36,39]. Three studies considered the influence of rinsing which could potentially wash off soap; the tails were rinsed with water between soap application and exposure to cercariae [28,39,43].

All these studies demonstrated that soap has the potential of protecting rodent tails against cercariae, and the soap protection can be represented by the reduction in worm burden in comparison with the control. As shown in Table 2, the protection varied among different soaps, which might be due to several influencing factors, such as soap concentration, the times of soap application, rinsing the tails or not, the rinse duration, and the time interval between soap application, tail rinse and tail exposure to cercariae.

**Table 2:** The protective effect of soap on rodent tails under different conditions.

Author (year)	Species	Soap name	Soap concentration	Soap use	Rinse		*X* time after (the last) soap use, tails were exposed to cercariae	Protection^a^ (%)
					Y/N	Duration		
Edungbola (1980) [36]	*S*. *mansoni*	Dettol	Undiluted	Once	No	-	24 h	100
			10^−1^ dilution					100
			10^−2^ dilution					100
			10^−3^ dilution					86.6
			10^−4^ dilution					62.6
			10^−5^ dilution					40.1
Fripp & Armstrong (1973) [39]	*S*. *mansoni*	Schistopel	-	4 d use 2 d break 2 d use	Yes	-	24 h	99.11
							48 h	97.87
							6 d	24.47
							8 d	22.34
				Once	Yes	-	2 h	98.96
							24 h	0
Katz *et al*. (1993) [28]	*S*. *mansoni*	Soaps containing essential oils	100 ppm	Once	No	-	Immediately	0–100
							24 h	0–100
		Eucalipto	100 ppm	Once	No	-	Immediately	29
							24 h	0
		Lux	100 ppm	Once	No	-	Immediately	85
							24 h	14
		F-44 (in laboratory)	1 ppm	Once	No	-	24 h	18
			10 ppm	Once	No	-	24 h	48
			100 ppm	Once	No	-	24 h	100
					Yes	30 min		100
					No	-	72 h	85
					Yes	30 min		0
					No	-	168 h	40
					Yes	30 min		0
		F-44 (in the field)	100 ppm	Once	Yes	-	Immediately	67
							24 h	58
							48 h	31
Santos Filho *et al*. (1987) [40]	*S*. *mansoni*	Soaps containing essential oils	100 ppm	Once	No	-	Immediately	68.2–100
							24 h	29.0–100
van Rensburg (1972) [43]	*S*. *matteei*	Schistopel	-	Once	Yes	3 min	Immediately	100
						8 min		98.15
						20 min		100
						40 min		100

^a^The protection (%) was the reduction in worms recovered from soap-treated mice/rats in comparison with the control.

Fripp and Armstrong investigated the protection from an experimental soap called Schistopel under different treatments [39]. They found that 2 hours after using this soap once, this soap provided rodent tails with almost 100% protection when exposed to cercariae for 20 minutes after the tail rinse, but this protection no longer existed when exposed to cercariae 24 hours after using soap, highlighting the influence of the time interval between washing and exposure to cercariae. Also, this soap was likely to have an accumulated protection since almost 100% protection was provided if using Schistopel on tails for 6 days and then exposing the tails to cercariae 48 hours after the last wash. This accumulative protective effect might be caused by the bacteriostatic agent in Schistopel, namely hexachlorophene, being absorbed by the skin tissues and not being removed immediately when washing. Another study investigated the effect of rinse duration on Schistopel protection and found that this soap was able to provide protection even after rinsing for 40 minutes [43].

Another two studies investigated the protective effect of soaps containing essential oils [28,40]. In those studies, the essential oils were extracted from fruits of the plant *Pterodon pubescens* and then incorporated into different soap formulations. Both studies found that these soaps protected mouse tails, but the protection varied among different soap formulations, ranging from 0–100% when tails were exposed to cercariae immediately after applying 100 ppm soap without rinsing the tails. This protection on tails was also inversely related to the time interval between soap application and exposure to cercariae. Among different soaps at 100 ppm containing different concentrations of essential oils and different proportions of other materials, F-44 showed the highest protection—100% even when mouse tails were washed for 30 minutes and exposed to cercariae 24 hours after soap application [28]. Its protection on tails increased with the increase in soap concentration, which was also found with the Dettol soap used by Edungbola, though tails were not rinsed in this study [36]. Also, no protection was obtained from F-44 when exposed to cercariae 72 h or 168 h after rinsing, but partial protection existed when tails were not washed [28], suggesting an influence of rinsing on reduced protection by soap.

Katz *et al*. also compared the efficacy of experimental soaps containing essential oils and two commercial soaps (Eucalipto and Lux) at reducing cercaria penetration and worm burden [28]. The results suggested that these two commercial soaps had less protective effect compared to those soaps containing essential oils. Eucalipto and Lux gave protection of 29% and 85% to tails immediately after soap application without rinsing, respectively, and the protection no longer existed for Eucalipto and dropped to only 14% for Lux 24 hours after applying soap [28]. However, Edungbola found that the Dettol soap at the dilution of 10^−2^ provided total protection under the same time interval [36].

A limitation of the findings presented in previous studies is that most these experimental studies were performed in controlled laboratory conditions. Only one study by Katz *et al*. conducted field experiments and compared with laboratory results [28]. There was a reduction in the protection on mouse tails in comparison with the laboratory results, dropping from 100% protection at 100 ppm immediately exposed to cercariae after applying soap in laboratory to 67% in the field. This reduced protection in the field might be because the water matrix in the field contained constituents which consumed the effective soap components when compared to the water used in the lab study.

## Conclusions

This systematic review demonstrates that soap has the potential to harm schistosome cercariae and provide skin with protection, thereby reducing the likelihood of infection during water-contact activities. Different adverse endpoints of cercariae were used in previous studies (i.e. mortality, motility, morphology, infectivity, viability), and several types of soap were tested (e.g. locally produced soap, commercial soap, experimental soap).

Soap’s toxicity to cercariae was associated with soap concentration and soap exposure time. However, for the studies on cercaria mortality, most studies did not mention how they evaluated the cercaria death and the number of cercariae used in these studies ranged from 10 to more than 300, which may lead to the differences in the reported results. Compared with the experiments that investigated the concentrations required to kill cercariae in water, lower soap doses were needed to make cercariae lose their capability of penetrating and developing into adult worms.

The protection provided by soap on the skin (represented by rodent tails) has also been revealed by several studies. However, it should be noted that the rodent tails were not rinsed in some experiments, which does not comply with the real-world scenario where rinsing is an essential step involved in almost every soap-related activity. High protection after rinsing was only found in two experimental soaps rather than commercial soaps, and no study investigated if protection on skin from commercial soaps could exist after rinsing, suggesting the importance of future research in this area.

The two protective aspects of soap both varied among different studies, which might be due to different cercarial endpoints assessed, soap ingredients, and experimental conditions (e.g. water characteristics, cercarial number, cercarial age). For skin protection, there are more influencing factors, such as the rinse duration, the skin-soap contact time, the amount of soap applied, the skin surface area exposed, and the time interval. However, most of the factors mentioned above have not been systematically studied and need further investigation.

Most studies in this review tested *S*. *mansoni*, and only two studies used *S*. *haematobium* and *S*. *mattheei*. Also, there are no studies using more than one species. Therefore, the sensitivity of different species of cercariae to soap remains unclear. In addition, only one study was carried out in the natural environment and it is unknown in some studies whether the snails used were laboratory-cultivated or naturally infected. Future studies need to conduct experiments using both cercariae produced by “wild” snails and water samples collected from disease-endemic areas with varying water qualities.

The effects of the plant endod and essential oils were investigated in a few studies. Endod showed a lower toxicity to cercariae compared with the commercial powder soaps, and there was a wide range of protection on mouse tails from different soaps containing essential oils and one soap formulation containing essential oils showed 100% protection even after rinsing the tails. Future studies could focus on incorporating plant extracts into soap to investigate whether the new product is effective at reducing the infection risk.

The potential benefit of soap as a schistosomiasis control measure is currently limited due to the low access to soap by people living in some schistosomiasis-endemic regions. According to the WHO, the proportion of the population with basic handwashing facilities including soap and water at home was only 4.83% in rural Ethiopia and 9.23% in rural Zambia in 2020 [47]. Because of the protective effects of soap that were demonstrated by many of the studies reviewed in this paper, the evidence supports that access to soap should be expanded in these regions.

Overall, however, there is insufficiently detailed knowledge about the impact of soap use against schistosome cercariae, which makes it difficult to propose reliable hygiene guidelines for preventing schistosomiasis, such as specific guidance on which soap types, amounts, and exposure times will be most protective. With the increasing awareness of the important role of WASH in schistosomiasis prevention, further research is needed to optimise the benefits of soap use and help to incorporate this as an intervention into multi-faceted schistosomiasis control strategies.

## Supporting information

S1 Methods Search methodology. Search terms and strategies of each database.
(DOCX)

S1 PRISMA 2020 checklist Systematic Review Checklist. (DOCX)

S2 PRISMA 2020 checklist Abstracts Checklist. (DOCX)

S1 Table Search results. Classification process and codes, a list of excluded and included studies, experimental setups and extracted data.
(XLSX)
